# Identification of six hub genes and two key pathways in two rat renal fibrosis models based on bioinformatics and RNA-seq transcriptome analyses

**DOI:** 10.3389/fmolb.2022.1035772

**Published:** 2022-11-09

**Authors:** Yueqin Cai, Jingan Chen, Jingyan Liu, Keyan Zhu, Zhixing Xu, Jianan Shen, Dejun Wang, Lisheng Chu

**Affiliations:** ^1^ School of Basic Medical Sciences, Zhejiang Chinese Medical University, Hangzhou, China; ^2^ Academy of Chinese Medical Sciences, Zhejiang Chinese Medical University, Hangzhou, China; ^3^ College of Pharmaceutical Sciences, Zhejiang Chinese Medical University, Hangzhou, China; ^4^ Laboratory Animal Resources Center, Westlake University, Hangzhou, China; ^5^ The First Medical College, Zhejiang Chinese Medical University, Hangzhou, China; ^6^ Department of Physiology, Zhejiang Chinese Medical University, Hangzhou, China

**Keywords:** renal fibrosis, RNA-seq, molecular mechanism, hub targets, drug prediction

## Abstract

Renal fibrosis (RF) is the common pathological manifestation and central treatment target of multiple chronic kidney diseases with high morbidity and mortality. Currently, the molecular mechanisms underlying RF remain poorly understood, and exploration of RF-related hub targets and pathways is urgently needed. In this study, two classical RF rat models (adenine and UUO) were established and evaluated by HE, Masson and immunohistochemical staining. To clear molecular mechanisms of RF, differentially expressed genes (DEGs) were identified using RNA-Seq analysis, hub targets and pathways were screened by bioinformatics (functional enrichment analyses, PPI network, and co-expression analysis), the screening results were verified by qRT-PCR, and potential drugs of RF were predicted by network pharmacology and molecular docking. The results illustrated that renal structures were severely damaged and fibrotic in adenine- and UUO-induced models, as evidenced by collagen deposition, enhanced expressions of biomarkers (TGF-β1 and α-SMA), reduction of E-cadherin biomarker, and severe renal function changes (significantly decreased UTP, CREA, Ccr, and ALB levels and increased UUN and BUN levels), etc. 1189 and 1253 RF-related DEGs were screened in the adenine and UUO models, respectively. Two key pathways (AGE-RAGE and NOD-like receptor) and their hub targets (Tgfb1, Col1a1, Nlrc4, Casp4, Trpm2, and Il18) were identified by PPI networks, co-expressed relationships, and qRT-PCR verification. Furthermore, various reported herbal ingredients (curcumin, resveratrol, honokiol, etc.) were considered as important drug candidates due to the strong binding affinity with these hub targets. Overall, this study mainly identified two key RF-related pathways (AGE-RAGE and NOD-like receptor), screened hub targets (Tgfb1, Col1a1, Nlrc4, Casp4, Trpm2, and Il18) that involved inflammation, ECM formation, myofibroblasts generation, and pyroptosis, etc., and provided referable drug candidates (curcumin, resveratrol, honokiol, etc.) in basic research and clinical treatment of RF.

## Introduction

Renal fibrosis (RF), associated with excessive accumulation of extracellular matrix (ECM) proteins and myofibroblasts, is a common consequence of various progressive kidney diseases and also the principal process of chronic kidney disease (CKD) to end-stage renal disease (Humphreys, 2018; Nastase et al., 2018). CKD is mainly caused by hypertension, diabetes, chronic pyelonephritis, autoimmune diseases, prolonged acute renal disease (Ammirati, 2020), and has been considered a serious public health problem with a global prevalence of 13.4% (11.7–15.1%) (Lv and Zhang, 2019). With such high morbidity of CKD, the risk of RF has been increasing and long-term RF causes irreversible damage to renal structure and function, resulting in increased mortality in patients with CKD and a great burden on society (Nogueira et al., 2017; Zhao et al., 2020). Studies have shown that the formation of RF exists as a complicated pathological mechanism involving inflammation, oxidative stress, apoptosis (Rayego-Mateos and Valdivielso, 2020). When the kidney injury occurs, inflammatory cells are rapidly recruited and release lots of profibrotic mediators (TNF-α, IL-6, and IL-1β, etc.), tubular epithelial cells and fibroblasts have to accumulate myofibroblasts and excessively product ECMs (collagens, fibronectin, proteoglycans, laminin, and endothelin), which finally cause glomerulosclerosis and tubular atrophy of RF (Liu, 2011; Meng, 2019). Nowadays, RF has joined the scientist’s attention and it is an urgent and unmet clinical need to understand the molecular pathogenesis of renal fibrosis.

Recently, the majority of molecular studies of RF are mainly concentrated in some common drug therapeutic targets (TGF-β1, CTGF, NOX4 and Smad3, etc.), as well as well-established RF-related pathways (HIF, TGF-β/Smad, PI3K-Akt and NF-κB pathways, etc.) (Liu, 2011; Liu et al., 2017; Rayego-Mateos and Valdivielso, 2020). For instance, TGF-β1 was recognized as a key fibrotic disease causative factor in RF progression, its specific up-regulation can induce the synthesis of ECM (collagens, proteoglycans, and glycoproteins) by activation of TGF-β/Smad pathway (Okuda et al., 1990; Hu et al., 2018). So far, though, the molecular mechanisms implicated in RF are still poorly understood, hence, it is of great importance to investigate more potential pathophysiological molecular mechanisms underlying the usual development of RF. To study the biopathology of RF, animal models are required. According to previous studies, the chemical toxicity of drugs and obstructive mechanical damage cause genetic alterations in the kidney, supporting the exploring of molecular and cellular mechanisms in RF pathogenesis. Unilateral ureteral obstruction (UUO) and adenine-induced chronic renal failure (ACRF) are two classic RF models that can respectively represent obstructive nephropathy and pharmacotoxic nephropathy (Kashioulis et al., 2018; Martínez-Klimova et al., 2019). These two animal models of RF have good reproducibility and have widely used in basic research of RF currently, and yet, their molecular mechanisms of RF at genomic levels need to be further investigated.

**FIGURE 1:**
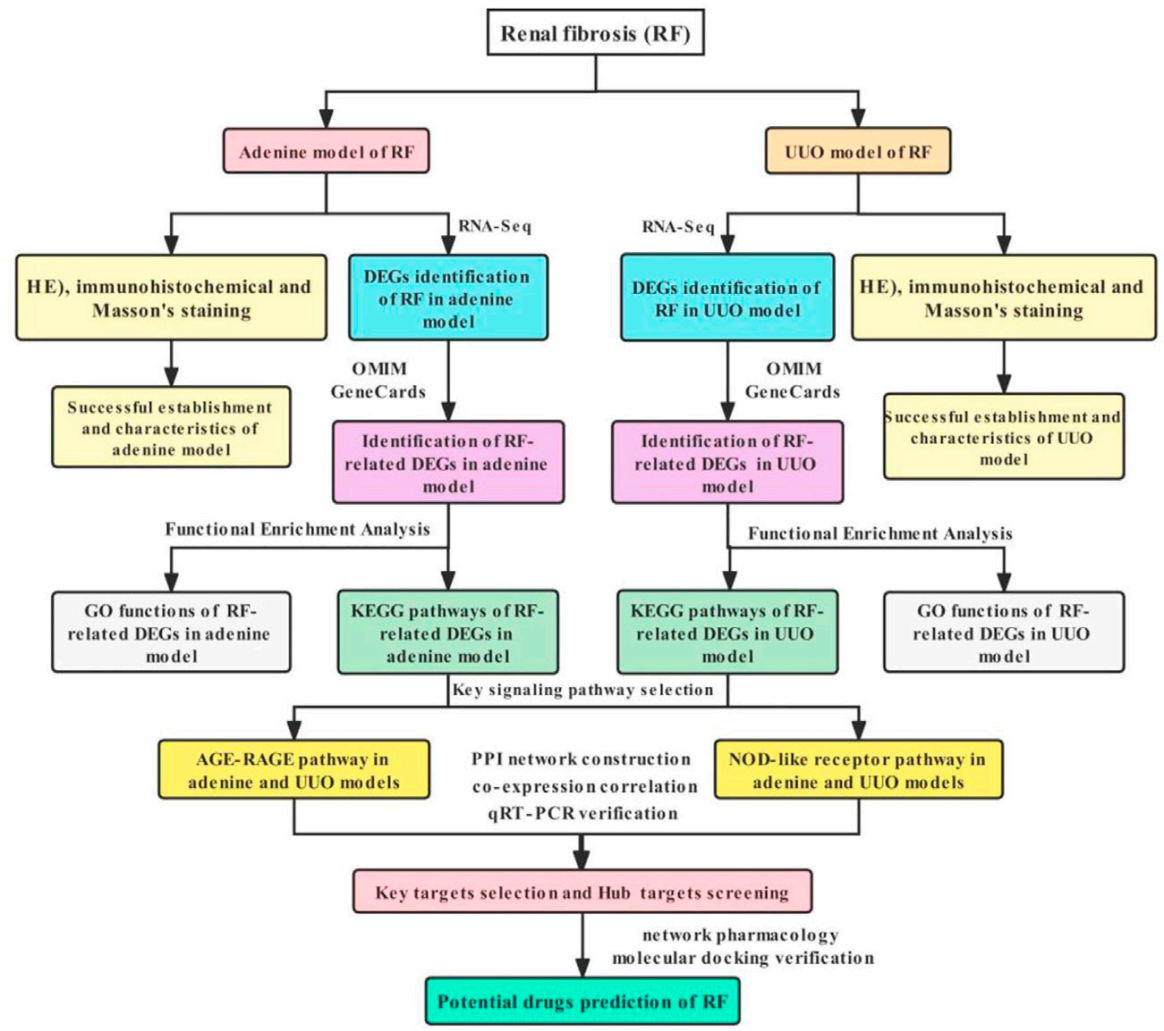
Workflow of exploring molecular mechanisms of RF based on RNA-Seq, bioinformatics, network pharmacology and molecular docking methods, including the adenine and UUO models.

Therefore, in this study, the adenine- and UUO-induced rat models of RF were established and their pathological changes were observed by hematoxylin-eosin (HE), immunohistochemical and Masson staining, differentially expressed genes (DEGs) were identified using RNA-Seq transcriptome analyses, and the protein-protein interaction (PPI) network, gene co-expression correlation analysis, Gene Ontology (GO) functions and Kyoto Encyclopedia of Genes and Genomes (KEGG) pathways of DEGs were further analyzed, the DEGs were verified by quantitative reverse transcription-polymerase chain reaction (qRT-PCR) and potential drug prediction of DEGs were performed by network pharmacology analysis and molecular docking, which might further increase a deeper understanding of potential molecular mechanisms of RF and offer more novel therapeutic targets, pathways and drug candidates against RF. All operation workflow were shown in Figure 1.

## Materials and methods

### Animal experimentation

60 Male SD rats (180–200 g, 8 weeks old) were purchased from SLAC Laboratory Animal Co. Ltd (Certificate No: SCXK (Shanghai) 2017-0005) and housed under standard environmental conditions (22 ± 2°C, 55–60% relative humidity, and 12 h light/ 12 h dark cycle) with free access to tap water and food in SPF grade animal room. All rats were kept for 7 days for proper acclimatization. Great care was taken to minimize their suffering and this study was approved by the Animal Ethics Committee of Zhejiang Chinese Medical University (Animal Ethics No: 20190902-04).

In this study, adenine (chemical) and UUO (surgical) models were established to investigate potential molecular mechanisms of RF as comprehensively as possible. In the establishment of the adenine model, 20 SD rats were randomized to adenine (*n* = 10, model) and control (*n* = 10, control) groups. During the experiments, 2.2 g adenine powder (Sigma-Aldrich, WXBC9818v, United States) was dissolved in 100 ml saline to prepare 2.2% adenine suspension. Rats in the adenine group were intragastrically administered with 220 mg/kg per day for 3 weeks. And in the control group, SD rats were given normal saline per day for 3 weeks. In the establishment of UUO model, 20 SD rats were randomly divided into UUO (n = 10, model) and sham (*n* = 10, control) groups. In the UUO group, with isoflurane anesthesia, UUO surgery was carried out and followed these steps: 1) the incision was made in the left abdominal cavity and the left ureter was exposed, 2) ligation of proximal and distal segments of the left ureter with 4–0 silk thread at both ends. 3) the ligated ureter was laid back in place and wounds were sutured. In the sham group, sham-operated rats were manipulated similarly, but their ureters were not ligated. After the operation, rats in two groups were fed with the conventional diet for 2 weeks. To observe renal function, 24-h metabolic changes of blood and urine of SD rats in the sham, UUO, control, and adenine groups were detected by HITACHI Automatic Aralyzer (Hitachi Limited, 3100, Japan), including urea total protein (UTP), urine urea nitrogen (UUN), urine creatinine (CREA), creatinine clearance (Ccr), serum albumin (ALB), blood urea nitrogen (BUN), and serum creatinine (Scr). At the end of the experiment, all SD rats were euthanized to collect their renal tissues for further experiments.

### Histopathology and immunohistochemistry

To evaluate the characteristics of the adenine and UUO models, hematoxylin and eosin (HE), Masson, and immunohistochemical staining were performed. Firstly, renal tissues fixed in 4% formalin were embedded in paraffin and then cut into 4 μm-thick sections. Then, in HE staining, sections were stained with hematoxylin and eosin using ST5010 Autostainer (Leica, Wetzlar, Germany). In Masson staining, sections were stained by Masson Stain Kit (G1340, Solarbio, China). In immunohistochemical staining, tissue sections were blocked with serum and incubated overnight at 4°C using the following primary antibodies: alpha-smooth muscle actin (α-SMA, 1: 50 dilution, ab5694, abcam, Cambridge, United Kingdom), E-cadherin (1: 50 dilution, ab1416, abcam, Cambridge, United Kingdom), and transforming growth factor-beta1 (TGF-β1, 1: 50 dilution, ab215715, abcam, Cambridge, United Kingdom). After primary antibody incubation, sections were incubated with a biotinylated secondary antibody (horseradish peroxidase, 1: 100 dilution) for 1 h at room temperature and then stained with 3,3′-diaminobenzidine tetrahydrochloride (DAB). Fourthly, all sections in HE, Masson, and immunohistochemical staining were photographed by a Nikon Eclipse 80i microscope (Nikon, Tokyo, Japan). Finally, quantitative analyses of Masson staining (collagen deposition) and immunohistochemistry (α-SMA, E-cadherin, and TGF-β1 staining density) were performed using Image-J software (Version 1.49, National Institutes of Health, Bethesda, United States) and the Image-Pro Plus software (Version 6.0, Media Cybernetics, Silver Spring, United States), respectively.

### RNA sequencing analysis

To explore potential molecular mechanisms of RF, RNA-seq analyses of the adenine and UUO models were performed. Firstly, three individual renal tissues of rats in the control, adenine, sham, and UUO groups were randomly selected to extract total RNAs using Trizol reagent (Invitrogen, Carlsbad, CA, United States), respectively. Then, the total RNA amount and purity of each sample was quantified using NanoDrop ND-1000 (NanoDrop, Wilmington, DE, United States). The RNA integrity was assessed by Bioanalyzer 2100 (Agilent, CA, United States) with RIN number >7.0, and confirmed by electrophoresis with denaturing agarose gel. Poly (A) RNA was purified from 1 μg total RNA using Dynabeads Oligo (dT) 25-61005 (Thermo Fisher, CA, United States) and fragmented into small pieces using Magnesium RNA Fragmentation Module (NEB, cat. e6150, United States). Then the cleaved RNA fragments were reverse-transcribed to create the cDNA by SuperScript™ II Reverse Transcriptase (Invitrogen, cat. 1896649, United States). The average insert size for the final cDNA library was 300 ± 50 bp. At last, DNA sequencing from the cDNA library was performed on an Illumina Novaseq™ 6000 (LC-Bio Technology CO., Ltd., Hangzhou, China) following the vendor’s recommended protocol.

### Data preprocessing and identification of DEGs

To identify DEGs of RF, the raw data from RNA-seq results in the adenine and UUO models were normalized and summarized to obtain clean reads. After removing the low-quality bases and undetermined bases, the reads were mapped and merged to reconstruct the comprehensive transcriptomes. After the final transcriptome was generated, the expression levels of all transcripts were estimated and gene expression levels were performed by calculating FPKM. The levels of each gene expression difference (fold change, FC) were calculated and compared with a false discovery rate (FDR) correction by using the “DESeq2″ package of R software (Version 4.1.1, Lucent Technologies, Bell Laboratories, New Jersey, United States). The genes conforming to |log2 FC| ≥ 2 and FDR value <0.05 were defined as DEGs and visualized by volcano plot and heatmap through “ggplot2″ package of R software (Version 4.1.1, Lucent Technologies, Bell Laboratories, New Jersey, United States).

### Identification of RF-related DEGs

To screen RF-related DEGs in the adenine and UUO models, RF-related genes were collected from the GeneCards (https://www.genecards.org/) and OMIM (https://omim.org/) databases. Then, DEGs in the adenine and UUO models were respectively intersected with RF-related genes to obtain their RF-related DEGs using the “VennDiagram” package of R software (Version 4.1.1, Lucent Technologies, Bell Laboratories, New Jersey, United States).

### Functional enrichment analysis

To further explore the underlying molecular mechanisms of RF, the biological functions and pathways of RF-related DEGs were analyzed. In biological functions, the gene ontology (GO) function of RF-related DEGs was analyzed using by the “org.Hs.eg.db”, “clusterProfiler,” “ggplot2″ and “enrichplot” packages of R software (Version 4.1.1, Lucent Technologies, Bell Laboratories, New Jersey, United States), including analyses of cellular component (CC), molecular function (MF), and biological process (BP). In pathways, Kyoto Encyclopedia of Genes and Genomes (KEGG) pathway enrichment analyses of RF-related DEGs were performed through the Metascape website (https://metascape.org/).

### Protein-protein interaction network construction and gene co-expression correlation analysis

To explore protein interaction and gene co-expression relationships among RF-related DEGs, PPI network and gene expression correlation analyses were performed. Firstly, PPI networks of RF-related DEGs in the adenine and UUO models were constructed using the STRING database (https://string-db.org/). The organism species was set as “Rattus norvegicus” with a correlation degree set as ≥ 0.40. Then, PPI networks were visualized by Cytoscape 3.8.2 software (Shannon et al., 2003). Moreover, in gene co-expression correlation analysis, the correlation plot was analyzed based on the Pearson correlation test in the ‘corrplot’ package of R software (Version 4.1.1, Lucent Technologies, Bell Laboratories, New Jersey, United States).

### Quantitative real-time PCR

To preliminarily verify the data of RNA-sequencing, a total of 24 renal samples (six samples in each group) from control, adenine, sham, and UUO groups were selected for qRT-PCR analysis, respectively. Firstly, total RNA was extracted from the renal sample by TRIzol reagents (Invitrogen, Carlsbad, CA, United States) following the manufacturer’s instructions. Then, reverse transcription of RNA to cDNA was performed with SuperScript™ IV Reverse Transcriptase reagents (ThermoFisher Scientific Inc, Waltham, MA, United States). Finally, cDNA was amplified by Applied Biosystems Step One Plus Real-Time PCR System (Thermo Fisher Scientific, Inc., MA, United States) following these procedures: a holding stage (95°C in one cycle of 30 s), a cycling stage (95°C in 40 cycles of 5 s and 60°C in 40 cycles of 30 s), and a melting curve stage (start at 55°C, increase by 0.5°C every 30 s until 95°C). All primer sequences were shown in Supplementary Table S1 and mRNA levels were calculated by the 2^−ΔΔCT^ method. Moreover, to ensure the accuracy of PCR validation, deviation plot of RF-related DEGs in RNA-seq was produced using the “ggpubr” package of R software (Version 4.1.1, Lucent Technologies, Bell Laboratories, New Jersey, United States).

### Drug prediction of RF-related DEGs

To offer drug candidates for the treatment of RF, drug prediction of RF-related DEGs were performed by network pharmacological analysis. Firstly, small molecule drugs and active ingredients of traditional Chinese medicine were collected from Drugbank (https://www.drugbank.ca/) and HERB (http://herb.ac.cn/) databases, respectively. Then, the “Disease-pathway-gene-drug” interaction network was constructed and visualized using Cytoscape 3.8.2 software (Shannon et al., 2003).

### Molecular docking analysis

To investigate the interaction and binding activities of targets with predicted drugs, molecular docking analysis was performed. All protein 3D structures of targets were downloaded from the PDB database (http://www.rcsb.org) and the ligand files of predicted drugs were obtained from the PubChem platform (http://pubchem.ncbi.nlm.nih.gov/), including Tgfb1 (PDB ID: 1KLA), Trpm2 (PDB ID: 6PUS), Trpv2 (PDB ID: 6U84), Agt (PDB ID: 5F9S), Nlrp3 (PDB ID: 7ALV), Il18 (PDB ID: 3WO2), Mapk10 (PDB ID: 3OY1), and Casp4 (PDB ID: 6NRY). After the optimization of the protein structure and small molecule ligands, molecular docking was completed by Autodock Vina 1.2.0 software (Eberhardt et al., 2021) and the binding effects were analyzed by Discovery studio visualizer software (Version 2020; BIOVIA, United States), including the docking conformation, binding energy, and intermolecular interactions (such as hydrophobicity, π-π stacking, hydrogen bonding, etc.).

### Statistical analysis

Statistical analyses were performed by SPSS software (Version 26.0, SPSS, Chicago, United States). One-way ANOVA test with least-significant difference (LSD) method was performed among three or more groups and all data were given as mean values ±standard deviation (SD). The results of *p* < 0.05 were considered statistically significant. Boxplots were produced by OriginPro Software (Version 2021; OriginLab, Northampton, MA, United States) and every point represented one sample.

## Results

### Functional, pathological and immunohistochemical characteristics of renal tissues in the adenine and UUO models

**FIGURE 2:**
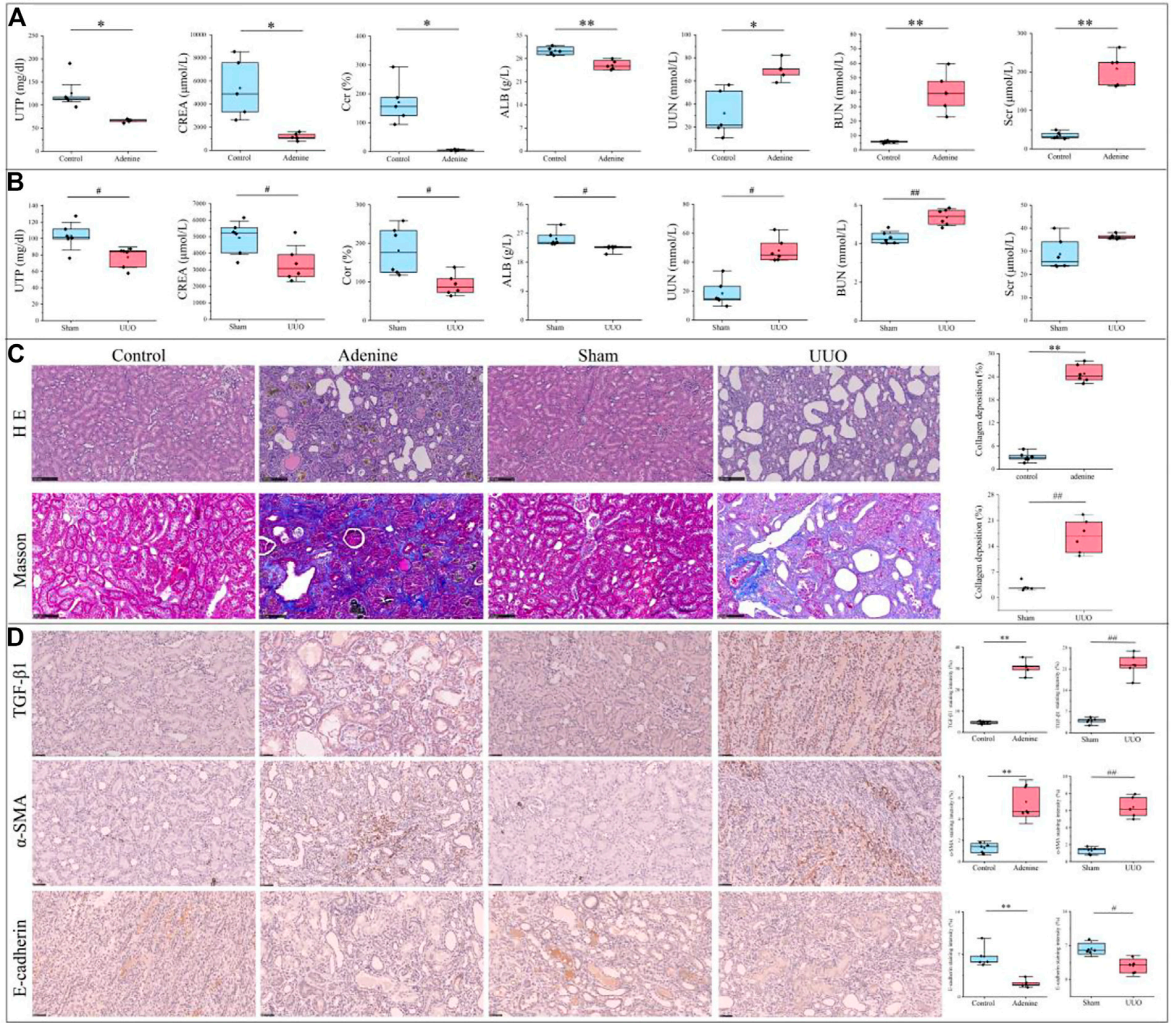
Renal function parameters, pathological characterization and immunohistochemical changes of RF-related adenine and UUO models. **(A)** Renal function parameters of rats in the control and adenine groups. **(B)** Renal function parameters of rats in the sham and UUO groups. **(C)** HE staining (200×), Masson staining (200×). and collagen deposition in the control, adenine, sham, and UUO groups. **(D)** Immunohistochemical staining (400×) and staining intensity of TGF-β, α-SMA, and E-cadherin in the control, adenine, sham, and UUO groups. All data showed mean ± SD, ^*^ *p* < 0.05 and ^**^ *p* < 0.01 compared to the control group, ^#^ *p* < 0.05 and ^##^ *p* < 0.01 compared to the sham group.

As shown in Figure 2A, renal function indices were significantly changed in the adenine group compared with the control group (*p* < 0.05 or *p* < 0.01 vs. control level), including downregulation of UTP, CREA, Ccr, and ALB levels and upregulation of UUN, BUN and Scr levels. In Figure 2B, renal function indices were significantly changed in the UUO group compared with the sham group (*p* < 0.05 or *p* < 0.01 vs. sham level), including downregulation of UTP, CREA, Ccr, and ALB levels and upregulation of UUN and BUN levels. In Figure 2C, renal morphology in the control group and sham group exhibited normal glomeruli, tubules, and interstitial tissues, in contrast, that in the adenine group and UUO group demonstrated tubular and glomerular atrophy, multifocal interstitial fibrosis, and lumen dilation. In Figure 2C, collagen deposition in the adenine group and UUO group was significantly higher than that in the control group (*p* < 0.05 vs. control level) and sham group (*p* < 0.05 vs. sham level), respectively. In Figure 2D, the protein expressions of TGF-β1 and α-SMA in the adenine group and UUO group were significantly higher than those in the control group (*p* < 0.05 vs. control level) and sham group (*p* < 0.05 vs. sham level). The protein expression of E-cadherin in the adenine group and UUO group was significantly lower than that in the control group (*p* < 0.05 vs. control level) and sham group (*p* < 0.05 vs. sham level). These findings suggested the successful establishment of two RF models (adenine and UUO).

### Identification of RF-related DEGs in the adenine and UUO models

**FIGURE 3:**
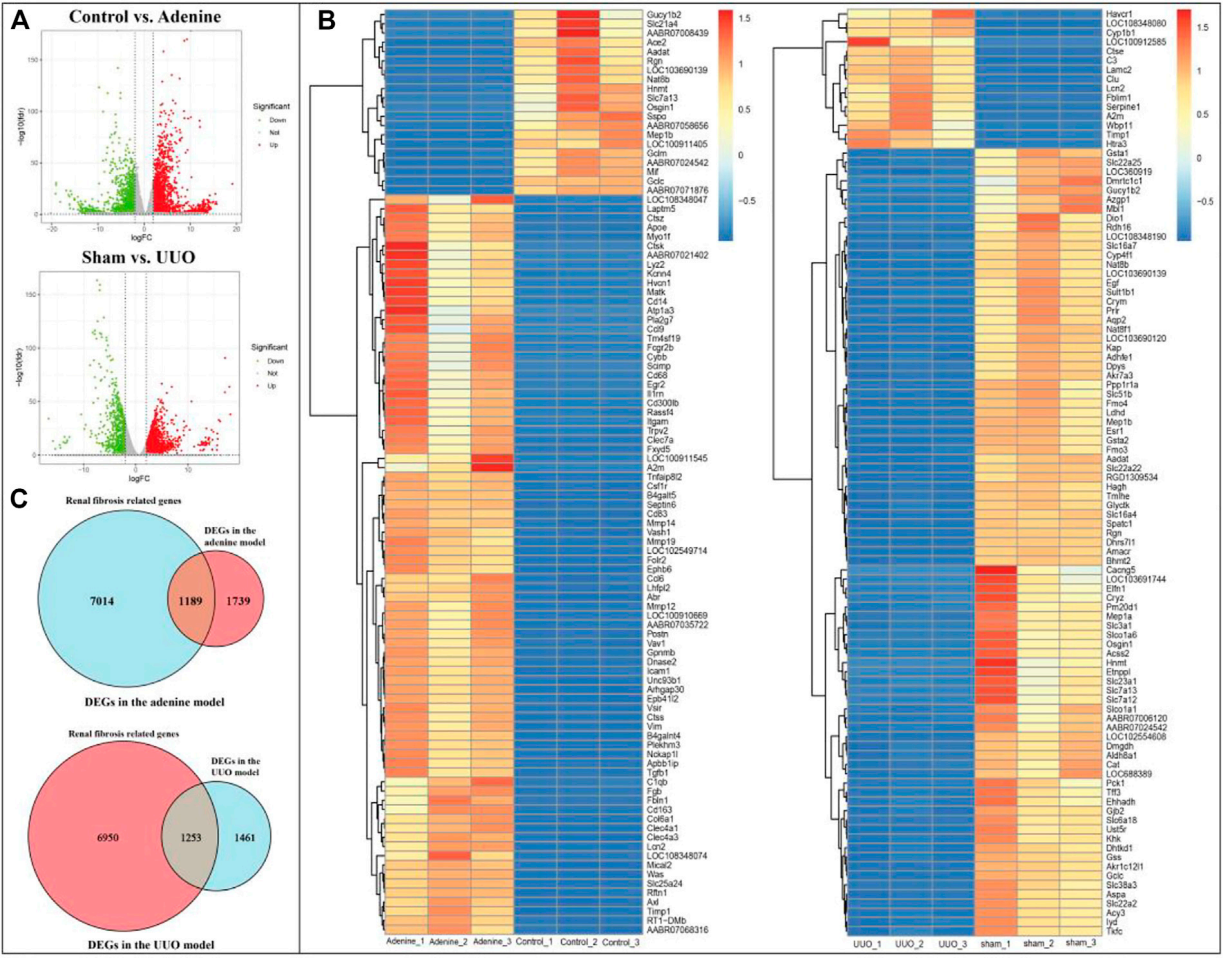
DEGs of RF in adenine and UUO models. **(A)** Volcanic maps showed DEGs in the adenine and UUO models, respectively. Red represented high expressed genes, green represented low expressed genes, and gray represented no different genes. **(B)** Heatmaps showed the top 100 DEGs with FDR value <0.05 in the adenine and UUO models, respectively. Red areas represented highly expressed genes and blue areas represented lowly expressed genes. **(C)** Venn diagram showed the intersection genes between RF-related genes and DEGs in the adenine and UUO models.

As shown in Figure 3A and Supplementary Table S2, according to the cutoff criteria of |log2 FC| > 2 and FDR <0.05, 2928 DEGs (2011 upregulated and 917 downregulated genes) in the adenine model and 2714 DEGs (2089 upregulated and 625 downregulated genes) in the UUO model were identified. In Figure 3B, the top 100 DEGs in the adenine model were obtained, including Tgfb1, Trpv2, A2m, and Trpv6, etc. And the top 100 DEGs in the UUO model were shown in the heatmap (Figure 3B), including Egf, Slc16a4, Slc22a2, and Slc16a10, etc. In Figure 3C, the overlap between DEGs in the adenine model and RF-related genes dataset contained 1189 RF-related DEGs, and the overlap between DEGs in the UUO model and RF-related genes dataset contained 1253 RF-related DEGs. Detailed information of DEGs in the adenine and UUO models was provided in Supplementary Table S2.

### Functional enrichment analyses of RF-related DEGs in the adenine and UUO models

**FIGURE 4:**
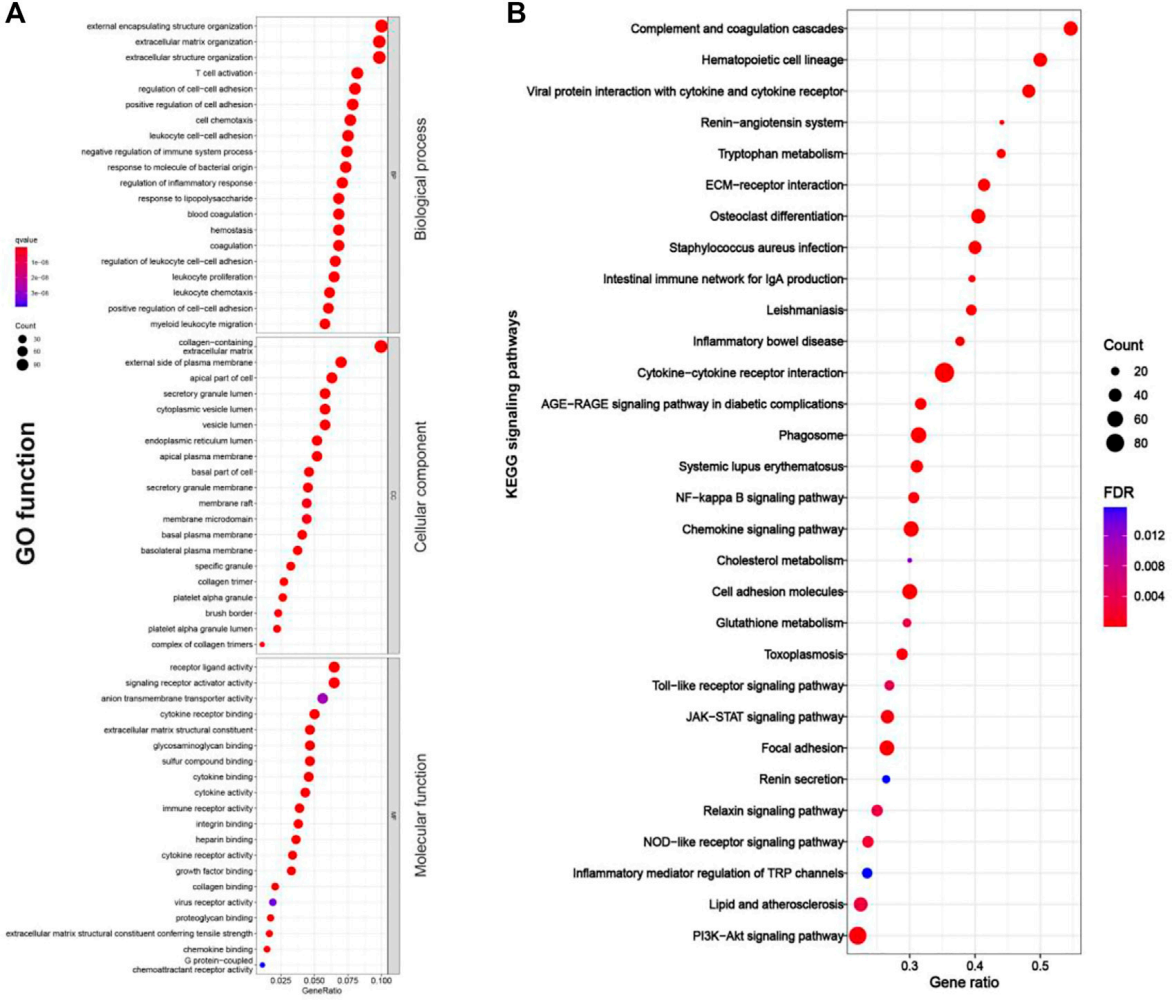
GO function and KEGG pathways enrichment results of RF-related DEGs in the adenine model. **(A)** Bubble charts of GO enrichment analysis, the *Y* axis displayed biological process, cellular component, and molecular function modules, the *X* axis represented gene ratio, count (the size of the nodes) indicated gene number, and qvalue (the intensity of the color) referred to -log10 *p*-value. **(B)** Bubble charts of 30 KEGG significant pathways with *p* < 0.05, the *Y*-axis displayed pathways, the *X*-axis represented gene ratio, count (the size of the nodes) indicated gene number, and FDR value (the intensity of the color) referred to -log10 *p*-value.

**FIGURE 5:**
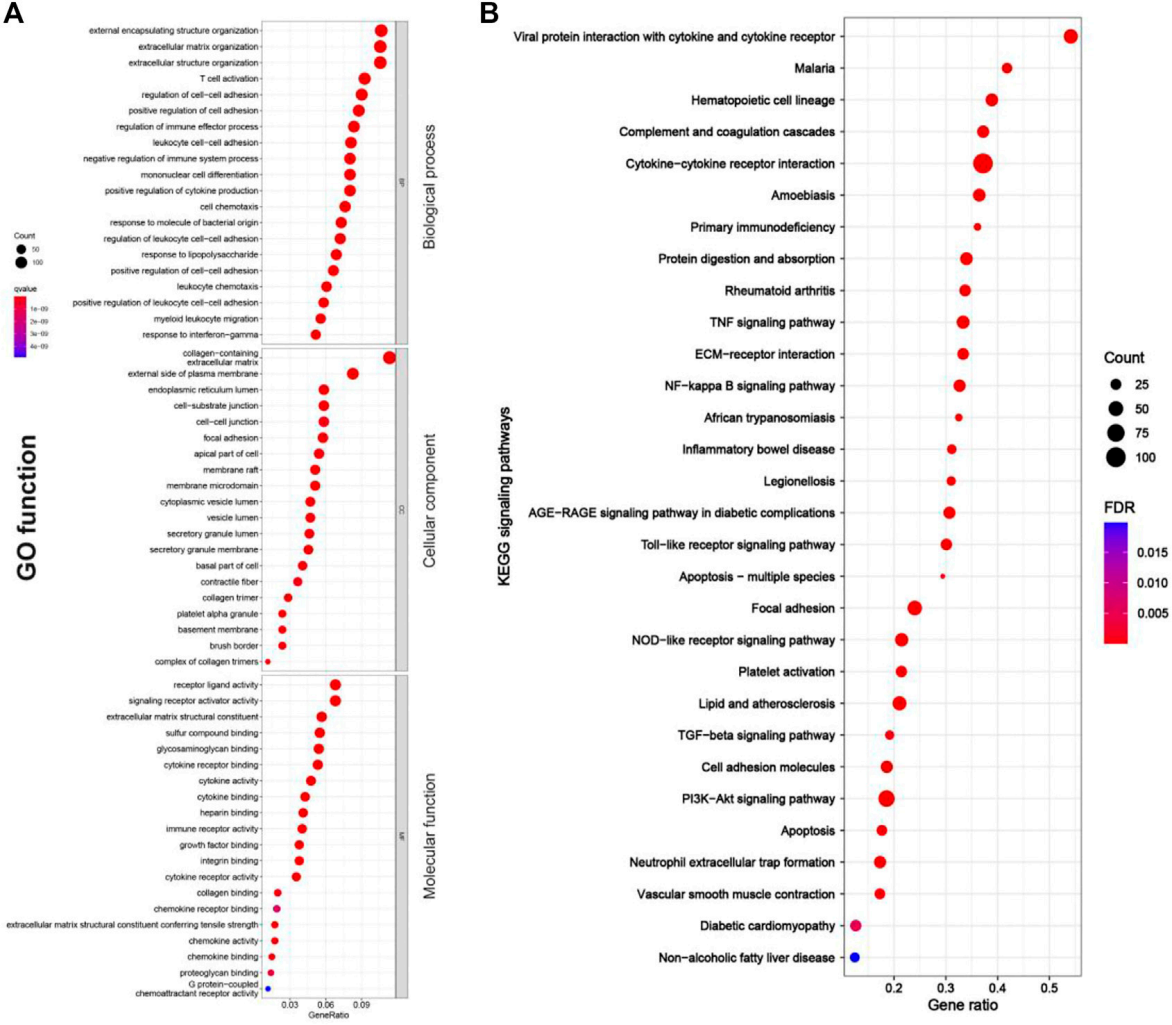
GO function and KEGG pathway enrichment results of RF-related DEGs in adenine model. **(A)** Bubble charts of GO enrichment analysis, the *Y* axis displayed biological process, cellular component, and molecular function modules, the *X* axis represented gene ratio, count (the size of the nodes) indicated gene number, and qvalue (the intensity of the color) referred to -log10 *p*-value. **(B)** Bubble charts of 30 KEGG significant pathways with *p* < 0.05, the *Y*-axis displayed pathways, the *X*-axis represented gene ratio, count (the size of the nodes) indicated gene number, and FDR value (the intensity of the color) referred to -log10 *p*-value.

As shown in Figures 4A, 5A, RF-related DEGs in the adenine and UUO models shared similar GO functions. Among them, the biological process module revealed that the RF-related DEGs were mainly enriched in external encapsulating structure organization, extracellular matrix organization, and positive regulation of cell adhesion, etc. The cellular component module showed that the RF-related DEGs were mainly involved in collagen−containing extracellular matrix, membrane raft, and external side of plasma membrane, etc. The molecular function module indicated that the RF-related DEGs were mainly associated with receptor ligand activity, extracellular matrix structural constituent, and collagen binding, etc. In Figures 4B, 5B, RF-related DEGs in the adenine and UUO models also shared similar KEGG pathways, including ECM-receptor interaction, NOD-like receptor pathway, AGE-RAGE pathway in diabetic complications, and Toll-like receptor pathway, etc. Detailed information of the GO and KEGG analyses was provided in Supplementary Tables S3, S4.

### Establishment and analysis of PPI networks

**FIGURE 6:**
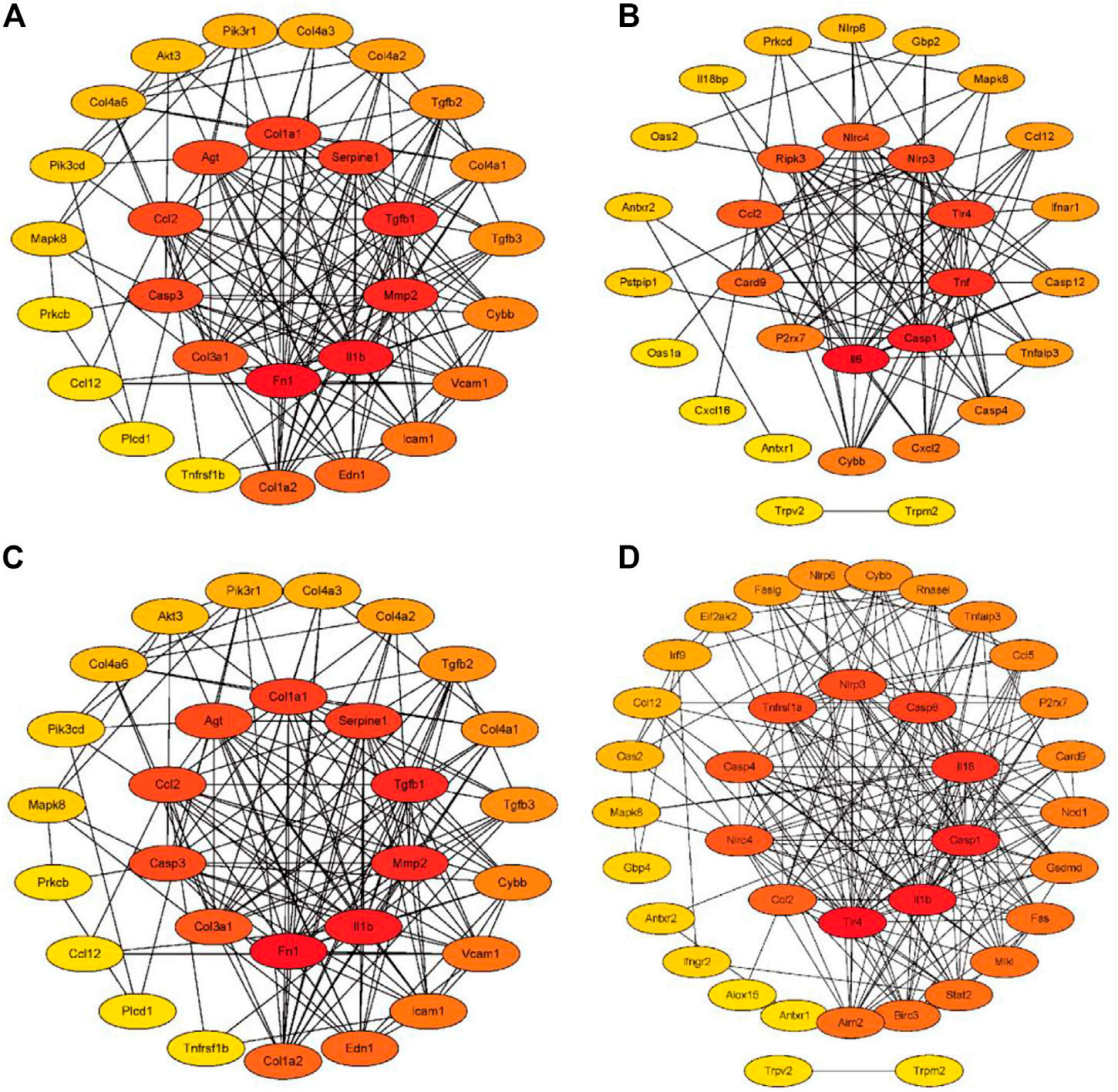
The PPI networks of RF-related DEGs in the adenine and UUO models. The intensity of the color from yellow to red indicated degree values (number of genetic links) from low to high, and top 10 RF-related DEGs were displayed in the center of the network. **(A)** The PPI network of RF-related DEGs from the AGE-RAGE pathway in the adenine model. **(B)** The PPI network of RF-related DEGs from NOD-like receptor pathway in the adenine model. **(C)** The PPI network of RF-related DEGs from the AGE-RAGE pathway in the UUO model. **(D)** The PPI network of RF-related DEGs from the NOD-like receptor pathway in the UUO model.

As shown in Figures 6A,B, 31 (AGE-RAGE pathway) and 30 (NOD-like receptor pathway) RF-related DEGs with protein interaction was found in the adenine model, respectively. In Figures 6C,D, 29 (AGE-RAGE pathway) and 37 (NOD-like receptor pathway) RF-related DEGs with protein interaction were found in the UUO model, respectively. According to the cytoHubba plugin’s degree ranking, in the adenine model, the top 10 RF-related DEGs were obtained in the AGE-RAGE pathway (Tgfb1, Col1a1, Il1b, and Agt, etc.) and NOD-like receptor pathway (Il6, Casp1, Nlrp3, and Nlrc4, etc.). In the UUO model, the top 10 RF-related DEGs were obtained in the AGE-RAGE pathway (Tgfb1, Col1a1, Il1b, and Agt, etc.) and NOD-like receptor pathway (Casp4, Il18, Nlrp3, and Nlrc4, etc.). Detailed information was provided in Supplementary Table S5.

### Correlation analysis of RF-related DEGs

**FIGURE 7:**
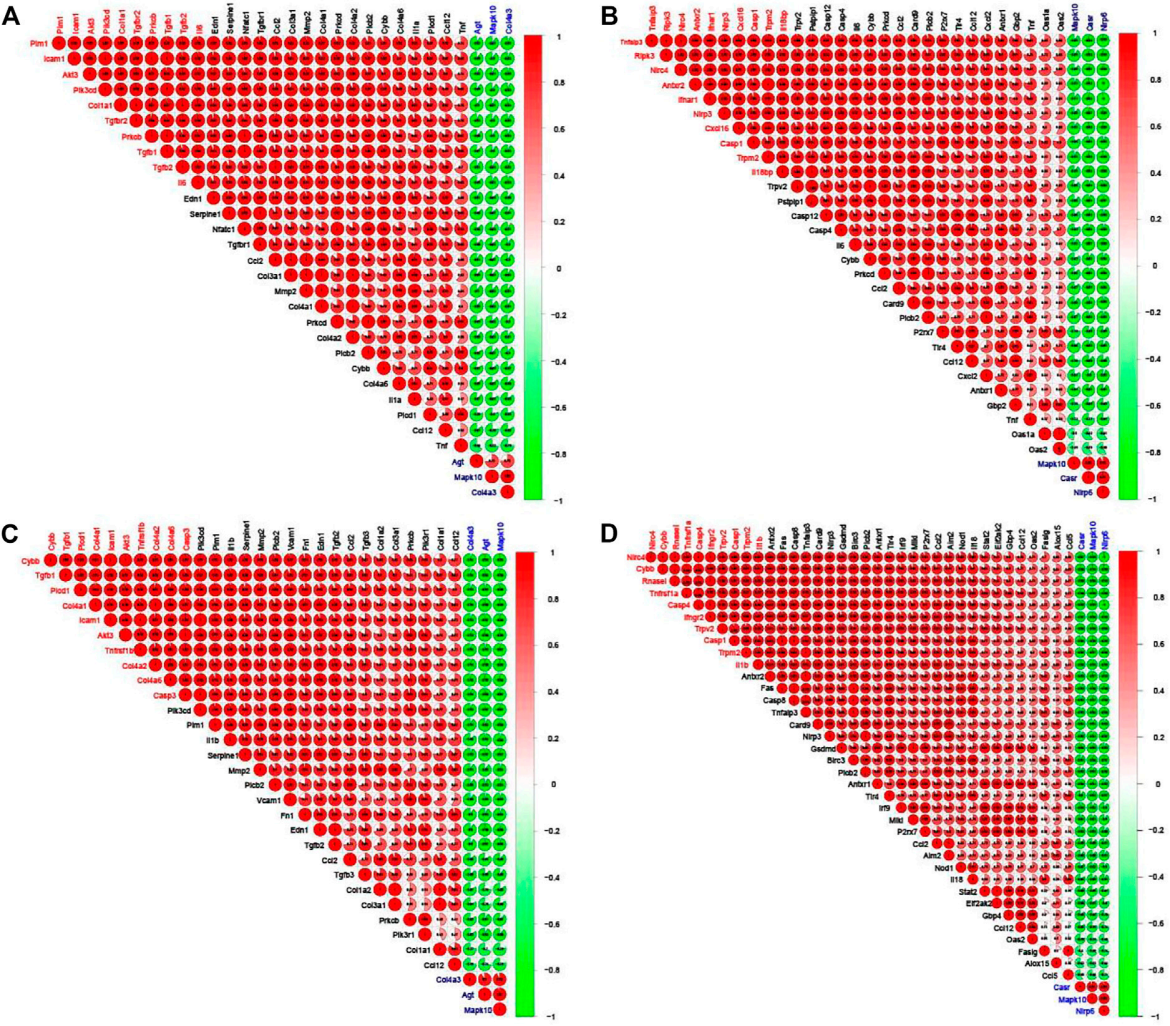
Corrplots of the correlation analysis between the RF-related DEGs. Red and green denoted positive and negative correlation, respectively. Every correlation coefficient was represented, and the darker the color, the higher the correlation coefficient. Top 10 DEGs with the number of positive correlation coefficient >0.7 were labeled red and top 3 DEGs with the number of negative correlation coefficient >0.7 were labeled blue. **(A)** The corrplot of RF-related DEGs from the AGE-RAGE pathway in the adenine model. **(B)** The corrplot of RF-related DEGs from NOD-like receptor pathway in the adenine model. **(C)** The corrplot of RF-related DEGs from the AGE-RAGE pathway in the UUO model. **(D)** The corrplot of RF-related DEGs from NOD-like receptor pathway in the UUO model.

As shown in Figure 7, in the adenine and UUO models, the highly positive or negative co-expressed relationships occurred among the RF-related DEGs in both AGE-RAGE pathway and NOD-like receptor pathway. Further screening by the gene number of correlation coefficient >0.7, in the adenine model, top 10 positive co-expressed DEGs (Tgfb1, Il6, Col1a1, and Pim1, etc.) and top 3 negative co-expressed DEGs (Agt, Mapk10, and Col4a3) were found in the AGE-RAGE pathway (Figure 7A), and top 10 positive co-expressed DEGs (Nlrc4, Trpm2, Nlrp3, and Il18, etc.) and top 3 negative co-expressed DEGs (Casr, Mapk10, and Nlrp6) were found in the NOD-like receptor pathway (Figure 7B). Moreover, in the UUO model, top 10 positive co-expressed DEGs (Tgfb1, Cybb, Icam1, and Casp3, etc.) and top 3 negative co-expressed DEGs (Agt, Mapk10, and Col4a3) were found in the AGE-RAGE pathway (Figure 7C), and top 10 positive co-expressed DEGs (Nlrc4, Casp4, Casp1, Trpm2, and Trpv2, etc.) and top 3 negative co-expressed DEGs (Casr, Mapk10, and Nlrp6) were found in the NOD-like receptor pathway (Figure 7D).

### Verification of the RF-related DEGs in the adenine and UUO models

**FIGURE 8:**
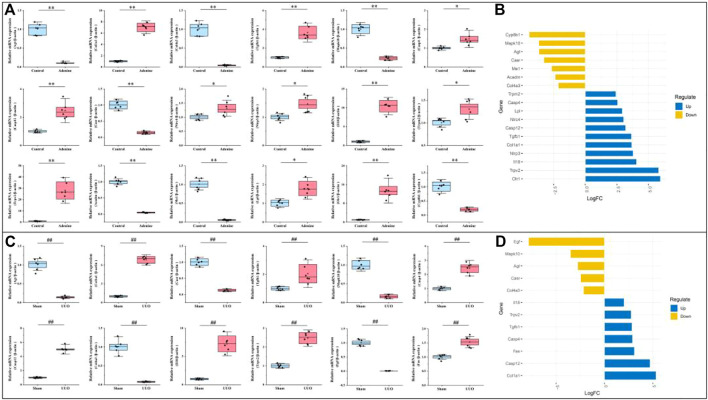
Verification of RF-related DEGs using qRT-PCR. **(A)** The gene expressions of Agt, Col1a1, Col4a3, Tgfb1, Mapk10, Casp4, Casp12, Casr, Nlrc4, Nlrp3, Il18, Trpm2, Trpv2, Acadm, Me1, Lpl, Olr1, and Cyp8b1 in the adenine model. **(B)** Deviation plot of RF-related DEGs in **(A)** was represented to ensure that gene expression trends in qRT-PCR and RNA-seq were consistent. **(C)** The gene expressions of Agt, Colia1, Col4a3, Mapk10, Tgfb1, Casp4, Casp12, Casr, Il18, Trpv2, Egf, Fas in the UUO model. **(D)** Deviation plot of RF-related DEGs in **(C)** was represented to ensure that gene expression trends in qRT-PCR and RNA-seq were consistent. Data showed mean ± SD, ^*^ *p* < 0.05 and ^**^ *p* < 0.01 compared to the control group, ^#^ *p* < 0.05 and ^##^ *p* < 0.01 compared to the sham group.

As shown in Figure 8A, compared with the control group, 11 DEGs (Col1a1, Tgfb1, Casp4, Casp12, Nlrc4, Nlrp3, Il18, Trpm2, Trpv2, Lpl, and Olr1) were significantly up-regulated and seven DEGs (Agt, Col4a3, Mapk10, Casr, Acadm, Me1, and Cyp8b1) were significantly down-regulated in the adenine group (*p* < 0.05 or *p* < 0.01 vs. control level), which was consistent with the result of DEGs expressed trends in RNA-seq (Figure 8B). In Figure 8C, compared with the sham group, seven DEGs (Colia1, Tgfb1, Casp4, Casp12, Il18, Trpv2, and Fas) were significantly up-regulated and five DEGs (Agt, Col4a3, Mapk10, Casr, and Egf) were significantly down-regulated in the UUO group (*p* < 0.05 or *p* < 0.01 vs. sham level), which was consistent with the result of DEGs expressed trends in RNA-seq (Figure 8D).

### Potential drug prediction of RF

**FIGURE 9:**
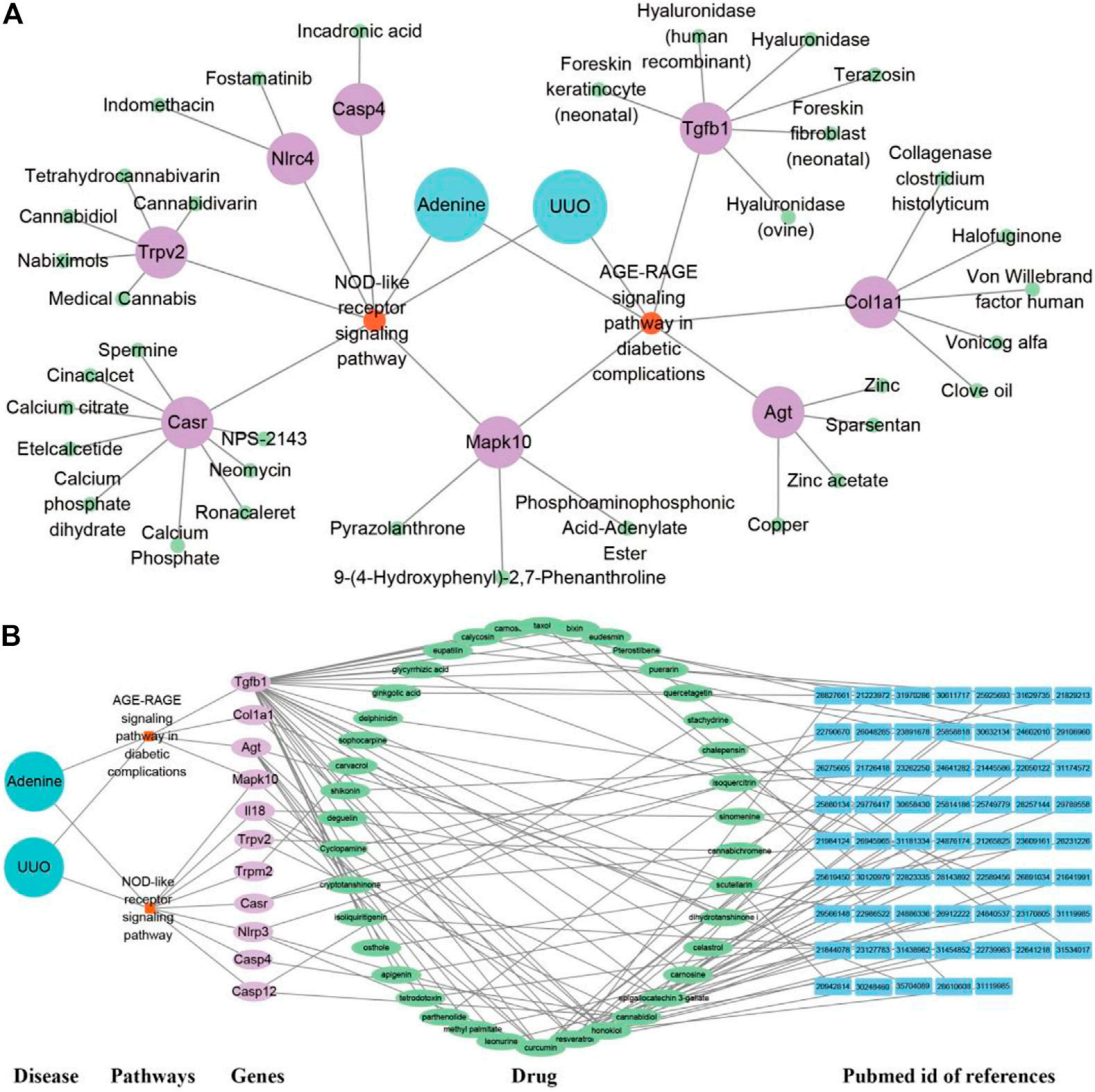
Potential drug prediction of renal fibrosis (RF) using network pharmacology analysis. **(A)** “Disease-pathway-gene-drug” interaction network of adenine and UUO models in the Drugbank database. The diseases were labeled by lake blue, pathways by orange, genes by purple, and drugs by green. **(B)** “Disease-pathway-gene-drug-reference” network of adenine and UUO models in the HERB database. The diseases were labeled by lake blue, pathways by orange, genes by purple, drugs by green, and PubMed id of references by blue.

As shown in Figure 9A, the drug network from the Drugbank database consisted of 47 nodes (two model nodes, two pathway nodes, eight drug target nodes, and 35 active drug nodes) and 96 edges. In both adenine and UUO models, RF-related DEGs (Tgfb1, Col1a1, Nlrc4, and Trpv2, etc.) in the AGE-RAGE pathway and NOD-like receptor pathway could be modulated by various drug candidates. Among them, Tgfb1 was modulated by six drugs (terazosin, foreskin fibroblast (neonatal), and hyaluronidase, etc.), Col1a1 by five drugs (halofuginone, vonicog alfa, and clove oil, etc.), and Nlrc4 by two drugs (Fostamatinib and Indomethacin). In Figure 9B, the TCM network from the HERB database consisted of 115 nodes (two model nodes, two pathway nodes, 11 drug target nodes, 39 active drug nodes, and 61 reference nodes) and 262 edges. In both adenine and UUO models, RF-related DEGs (Tgfb1, Col1a1, Il18, Casp4, and Trpm2, etc.) in the AGE-RAGE pathway and NOD-like receptor pathway could be modulated by various drug candidates. Among them, Tgfb1 was modulated by thirty drugs (resveratrol, taxol, puerarin, and quercetagetin, etc), Col1a1 by two drugs (dihydrotanshinone I and honokiol), Il18 by four drugs (curcumin, sinomenine, honokiol, and epigallocatechin 3-gallate), Casp4 by cannabidiol, and Trpm2 by carvacrol. Detailed information was shown in Supplementary Table S6.

### Verification of “targets-predicted drugs” interaction by molecular docking

**FIGURE 10:**
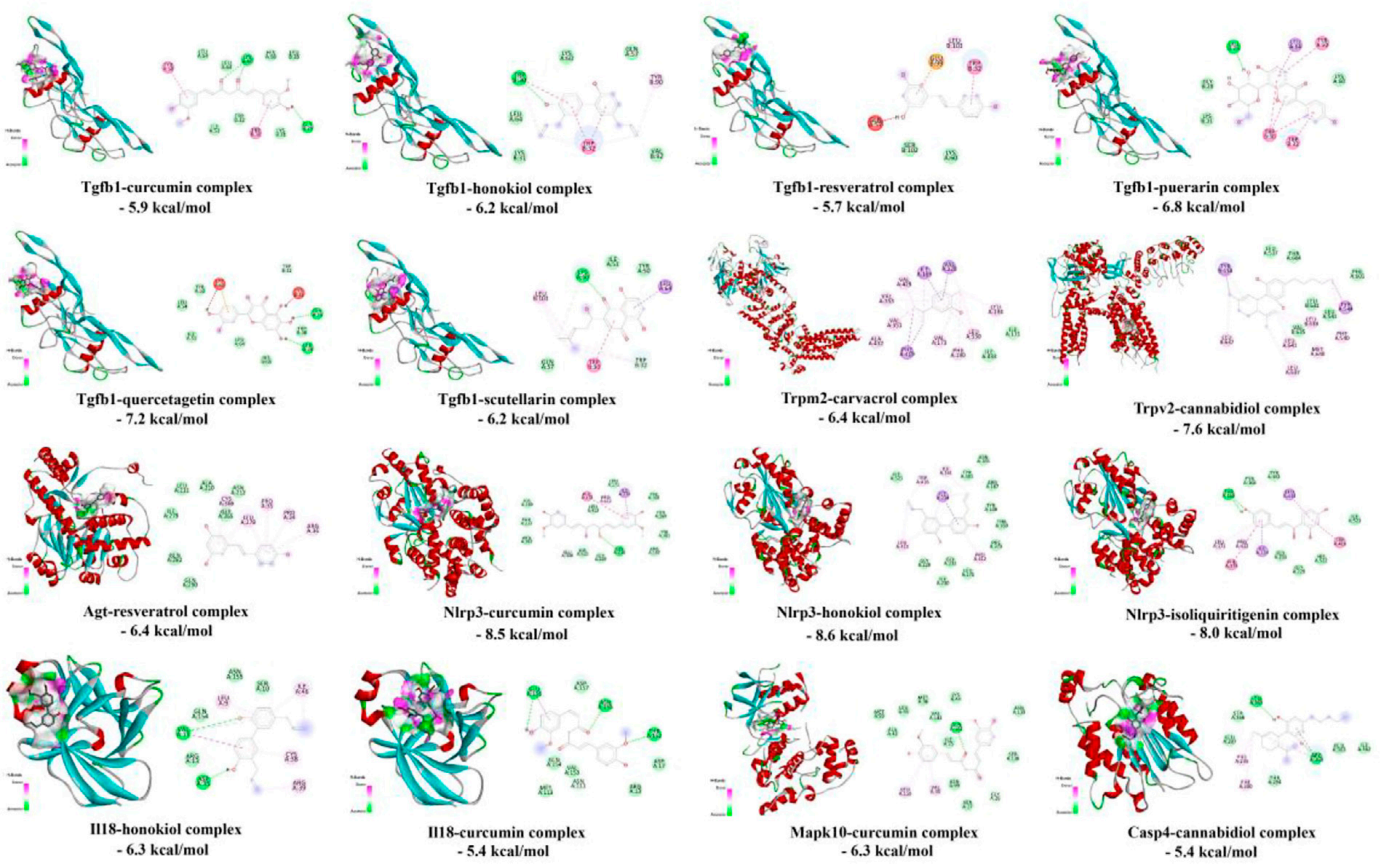
Molecular docking analysis of hub targets and ingredients.

As shown in Figure 10, the affinity energies of all protein-ligand complexes were the stable conformation due to binding free energies < -5 kcal/mol. Furthermore, 2D diagrams cleared that these targets and herbal ingredients in the protein-ligand complexes existed the strong non-bond interactions that regulated ligand binding and protein activity, involving the formation of hydrogen bonding, hydrophobic bond, van der waals, π-π interaction, and π-alkyl interaction etc.

## Discussion

Renal fibrosis (RF) is a disease with the pathological characterization of glomerulosclerosis and tubulointerstitial fibrosis in the clinic and common outcome of various chronic kidney diseases caused by diabetes mellitus, obesity, HIV infection, hypertension, etc. (Liu, 2011; Humphreys, 2018). It is widely considered that the inhibition of RF plays a critical role in improving the prognosis of all chronic kidney diseases and delays the process of end-stage renal disease, which causes the explosion of molecular mechanisms and treatment strategies of RF (Liu, 2011). Therefore, the investigation of molecular mechanisms in RF is crucially important to understand the pathological mechanisms of RF and offers more options (new investigative targets and pathways) in drug research of RF. The *in vivo* results indicated that both adenine- and UUO-induced rat models were successfully built, as demonstrated by severe renal function changes (significantly decreased UTP, CREA, Ccr, and ALB levels and increased UUN and BUN levels), glomerulosclerosis, lumen dilation, collagen deposition, enhanced expressions of biomarkers (TGF-β1 and α-SMA), and reduction of E-cadherin biomarker (Figure 2). RNA-seq results presented that 1189 and 1253 RF-related DEGs were identified in the adenine and UUO models, respectively (Figure 3C). GO analysis (Figures 4, 5) showed that RF-related DEGs were mainly enriched in various functions (collagen binding, collagen−containing extracellular matrix, and cell adhesion, etc.) that were associated with ECM production in RF. And KEGG analysis (Figures 4, 5) indicated that adenine and UUO models existed with 95 and 109 pathways, respectively, in which AGE-RAGE and NOD-like receptor pathway existed potential research value in RF due to their correlation with renal cells injuries (fibrosis, inflammation and apoptosis) in CKD (Yang et al., 2019) but unclear roles in RF-related reports until now. After screening by PPI and gene co-expressed relation (Figures 6, 7), top RF-related DEGs (Tgfb1, Col1a1, Mapk10, Nlrc4, Nlrp3, Casp4, Trpm2, Trpv2, and Il18, etc.) in these two pathways were initially identified as key targets due to the further verification by qRT-PCR (Figure 8).

As the important members of AGE-RAGE pathway, Tgfb1 was known as the predominant profibrotic factor that drived glomerular and tubulointerstitial fibrosis in different types of kidney diseases (López-Hernández and López-Novoa, 2012), and Col1a1 was a crucial fibrotic gene in ECM formation and fibroplasia of RF (Hosper et al., 2013). Previous works presented that overexpressed Tgfb1 could induce upregulation of collagen genes (Col1a1, Col3a1, and Col4a1, etc.) to stimulate ECM accumulation (collagens, fibronectin, and endothelin, etc.) in various renal cells (fibroblasts, tubular cells, glomerular mesangial cells and epithelial cells, etc.) (Livingston et al., 2022), which was mainly associated with the activation of TGF-β/Smad and AGE-PAGE pathways (De Vriese et al., 2003; Ma and Meng, 2019). In this study, RNA-seq (Supplementary Table S2) and qRT-PCR (Figure 8) results also demonstrated that mRNA levels of Tgfb1 and Col1a1 were significantly increased in both adenine and UUO models of RF, and immunohistochemical analysis (Figure 2D) showed that protein expression of TGF-β1 was significantly increased. According to these results and previous studies (De Vriese et al., 2003; Meng et al., 2016; Ma and Meng, 2019), it was hypothesized that overexpressed Tgfb1 could bind to the plasma membrane receptor (like Tgfbr1), trigger Smad2/3 phosphorylation, promote formation of a transcription complex (Smad2/3-Smad4) that translocated to the nucleus, and further upregulated downstream gene expressions (like Col1a1), which activated TGF-β/Smad and AGE-RAGE pathways of RF and promoted collagen accumulation (Figure 2C), myofibroblast generation with overexpressed α-SMA (Figure 2D) and epithelial-mesenchymal transition (EMT) with underexpressed E-cadherin (Figure 2D). Tgfb1 and Col1a1 were closely related to the occurrence and progression of RF, as a result, had become the hub targets of AGE-RAGE pathway.

Moreover, in the NOD-like receptor pathway, RF-related DEGs (Nlrc4, Nlrp3, Casp4, Trpm2, Il18, and Il1b, etc.) were significantly upregulated (Supplementary Table S2), all of which were reported to be associated with pyroptosis (Zhang and Wang, 2019; Wen et al., 2022). Among them, Nlrc4 served as a sensor for Casp1 activation, promotes Casp1-mediated pyroptosis of HK-2 cells, and aggravated RF in diabetic animals by accelerating inflammatory cell infiltration and pyroptosis-associated protein (gsdmd and caspase-1) expression (Wen et al., 2022). Casp4 was related to activation of inflammatory response, tubular injury and interstitial fibrosis under pathological conditions of RF, and its inhibition significantly blunted TGF-β1, fibronectin, and collagen I expressions in the obstructed kidney of UUO mice (Miao et al., 2019). Il18 played an important role in the progression of RF *via* modulating inflammation cells infiltration, myofibroblasts formation, and the expression of inflammatory cytokines and chemokines, etc. (Liang et al., 2018). Trpm2 ablation significantly attenuated RF in UUO mice *via* inhibiting TGF-β1-induced fibrosis and inflammation, accompanied by the reduction of fibrotic genes such as α-SMA and Col1α1 (Wang et al., 2019). In this study, qRT-PCR (Figure 8) further confirmed that mRNA levels of RF-related DEGs (Nlrc4, Casp4, Il18 and Trpm2, etc.) were markedly increased in the NOD-like receptor pathway, and thus pathologic process of RF might be driven by a pyroptosis-related mechanism. Furthermore, Nlrc4, Casp4, Il18 and Trpm2 were expected to become hub targets of NOD-like receptor pathway.

Obviously, AGE-RAGE and NOD-like receptor pathways played a crucial role in the pathological process and treatment of RF, involving in triggering inflammation, ECM formation (collagen, elastin, and fibronectin), myofibroblasts transdifferentiation, and pyroptosis, etc. Moreover, in the adenine and UUO models, a highly positive gene co-expressed relation (correlation coefficient >0.8) occurred between Tgfb1 and Col1a1 in the AGE-RAGE pathway (Figures 7A,C), among Nlrc4, Casp4, Il18 and Trpm2 in the NOD-like receptor pathway (Figures 7B,D), which was confirmed by qRT-PCR (Figure 8). Collectively, these six RF-related DEGs and two pathways were finally identified as hub therapeutic targets in basic drug research of RF.

Furthermore, this study offered various potential drug candidates for the treatment of RF (Figures 9, 10). In Figure 9, as an important target, Tgfb1 could be treated by various drug candidates, including curcumin, resveratrol, honokiol, cannabidiol, etc. Among them, curcumin could significantly decrease expression levels of Tgfb1, weaken phosphorylation of Smad2 and Smad3, PI3K, AKT, and NF-κB to inhibit TLR4/NF-κB, PI3K/AKT and TGF-β/Smads pathway, which attenuated EMT, the inflammatory response and tubulointerstitial fibrosis of UUO rats (Li et al., 2011; Wang et al., 2020). Resveratrol excellently curbed the process of RF by decreasing Tgfb1 and antagonizing the hedgehog pathway to inhibit EMT process and ECM deposition in UUO rats (Bai et al., 2014). Honokiol ameliorated RF by suppressing the secretion of pro-fibrotic factors (Col1a1, Tgfb1, and fibronectin) (Chiang et al., 2011). Meanwhile, curcumin, resveratrol, or honokiol might stably bind to the active pockets of Tgfb1, Casp4, Agt, Col1a1, and Il18 and regulated their activity by the strong non-bond interactions (Figure 10). Herein, the top three drug candidates (curcumin, resveratrol, and honokiol) has been extensively studied in RF and seemed to helpfully ameliorate RF by synergistically regulating gene expression levels or protein activation of Tgfb1, Agt, Col1a1, and Il18, and then effectively inhibiting activation of AGE-RAGE and NOD-like receptor pathways.

Certainly, the roles of hub targets (Tgfb1, Col1a1, Nlrc4, Casp4, Il18 and Trpm2) and pathways (AGE-RAGE and NOD-like receptor) were not fully elucidated, and their identification was just the beginning of exploring the internal complex mechanisms of RF. Moreover, the other RF-related DEGs (Trpv2, Olr1, Mapk10, and Agt, etc.) also had potential research values in exploring molecular mechanisms of RF. For example, Trpv2 was top 100 DEGs in heatmap (Figure 3B) and its mRNA levels were increased by 1-fold in the adenine model and 23-fold in the UUO model (Figure 8). However, the roles of these DEGs in molecular mechanisms of RF are not reported and thus not temporarily classified as hub targets in this study. In our future work, it is hope that more hub targets and pathways will be explored and screened by various research techniques, including the establishment of more animal models, expansion of the sample size and scope of sequencing (miRNA, lncRNA, circRNA), and bioinformatics methods like weighted gene co-expression network analysis, etc.

## Conclusion

This study provided six hub therapeutic targets, two key pathways, and various predicted drugs for the basic research and clinical treatment of RF. The *in vivo* data indicated that glomerulosclerosis, severe collagen deposition, enhanced expressions of biomarkers (TGF-β1 and α-SMA), reduction of E-cadherin biomarkers, and significant renal function changes (significantly decreased UTP, CREA, Ccr, and ALB levels and increased UUN and BUN levels) occurred in both adenine- and UUO-induced models. RNA-seq data exhibited that 1189 and 1253 RF-related DEGs were identified in the adenine and UUO models, respectively. Bioinformatics analysis illustrated that two key pathways (AGE-RAGE and NOD-like receptor) might exert a pivotal role in pathological process and treatment of RF by affecting inflammation, collagen synthesis, EMT process and pyroptosis of various renal cells. In these pathways, RF-related DEGs existed the strong protein interactions and gene co-expression relationships, and six RF-related DEGs (Tgfb1, Col1a1, Nlrc4, Casp4, Trpm2, and Il18) were considered as hub therapeutic targets. Furthermore, a quantity of reported herbal ingredients (curcumin, resveratrol, honokiol, etc.) were predicted to co-regulate many RF-related DEGs like Tgfb1 and inhibit AGE-RAGE and NOD-like receptor pathways to improve RF and delay the progression of CKD to ESRD. Further studies are required to investigate and verify the molecular mechanisms of RF, especially the AGE-RAGE and NOD-like receptor pathways. Taken together, we analyzed the pathological and molecular mechanisms in two RF models (adenine and UUO), which provided a direction and shed light on basic and drug research of RF.

## Data Availability

The data presented in the study are deposited in the GEO database (accession number: GSE216376).

## Abbreviations

Casp4, caspase 4; Col1a1, collagen alpha-1chain, Alpha-1; Il18, interleukin-18Tgfb1, transforming growth factor beta-1; Nlrp3, NOD-like receptor thermal protein domain associated protein 3; Nlrc4, NLR family CARD domain-containing protein 4; Trpm2, transient receptor potential cation channel subfamily M member 2.
